# A Portable Smart-Phone Readout Device for the Detection of Mercury Contamination Based on an Aptamer-Assay Nanosensor

**DOI:** 10.3390/s16111871

**Published:** 2016-11-08

**Authors:** Wei Xiao, Meng Xiao, Qiangqiang Fu, Shiting Yu, Haicong Shen, Hongfen Bian, Yong Tang

**Affiliations:** 1 Department of Bioengineering, Guangdong Province Key Laboratory of Molecular Immunology and Antibody Engineering, Jinan University, Guangzhou 510632, China; xkevent@stu2014.jnu.edu.cn (W.X.); cf412xm@stu2015.jnu.edu.cn (M.X.); 1423221001@stu2014.jnu.edu.cn (Q.F.); Shiting@stu2014.jnu.edu.cn (S.Y.); alan@stu2015.jnu.edu.cn (H.S.); alexis@stu2015.jnu.edu.cn (H.B.); 2 Institute of Food Safety and Nutrition, Jinan University, Guangzhou 510632, China; 3 Institute of Biotranslational Medicine, Jinan University, Guangzhou 510632, China

**Keywords:** smartphone, microwell reader, mercury ions, colorimetric aptamer nanosensor, gold nanoparticle

## Abstract

The detection of environmental mercury (Hg) contamination requires complex and expensive instruments and professional technicians. We present a simple, sensitive, and portable Hg^2+^ detection system based on a smartphone and colorimetric aptamer nanosensor. A smartphone equipped with a light meter app was used to detect, record, and process signals from a smartphone-based microwell reader (MR S-phone), which is composed of a simple light source and a miniaturized assay platform. The colorimetric readout of the aptamer nanosensor is based on a specific interaction between the selected aptamer and Hg^2+^, which leads to a color change in the reaction solution due to an aggregation of gold nanoparticles (AuNPs). The MR S-phone-based AuNPs-aptamer colorimetric sensor system could reliably detect Hg^2+^ in both tap water and Pearl River water samples and produced a linear colorimetric readout of Hg^2+^ concentration in the range of 1 ng/mL–32 ng/mL with a correlation of 0.991, and a limit of detection (LOD) of 0.28 ng/mL for Hg^2+^. The detection could be quickly completed in only 20 min. Our novel mercury detection assay is simple, rapid, and sensitive, and it provides new strategies for the on-site detection of mercury contamination in any environment.

## 1. Introduction

Mercury is one of the most toxic and harmful elements to human health [1,2,3]. Through mining, solid waste incineration, and the combustion of fossil fuels, mercury has leached into water, air, and soil [4,5,6,7]. Mercury accumulates in the human body through food and drinking water taken from Hg(II)-contaminated sources such as irrigated farmland and polluted bodies of water. Mercury accumulation in the body leads to brain and liver damage [8,9]. Therefore, there is a great need for simple, reliable on-site analyses for Hg^2+^ in surface water and could potentially prevent mercury-related illness through the early detection of environmental contamination. A series of spectroscopic methods have been used to measure trace concentrations of Hg^2+^ in environmental samples, such as atomic absorption spectroscopy (AAS) [10,11], inductively coupled plasma mass spectrometry (ICP-MS) [12,13], and atomic fluorescence spectrometry (AFS) [14]. While these assays are highly sensitive, selective, and accurate, they require complex sample preparation procedures, expensive and bulky instruments, and professionally trained personnel to run the tests. Many of these tests are also not portable, and samples need to be collected and returned to a lab for analysis. Therefore, they are limited in use and not well suited for rapid on-site detection of mercury.

Recent advances in biosensors have shown great promise for using portable heavy metal chromium assays in resource-limited environments [15,16,17,18]. Electrochemical biosensors [19,20], plasmon-enhanced photoelectrochemical sensors [21], surface-enhanced Raman spectroscopy (SERS)-based biosensors [22], colorimetric biosensors [23,24], and fluorescent sensors [25,26] have been reported as alternative approaches for mercury ion detection, providing high sensitivity, specificity, and ease of signal read-out. However, these reported systems are still difficult to be used in resource-limited environments due to their relatively complex operations and the requirement for expensive or bulky instruments, such as UV-visible (UV-vis) spectrometers, fluorescence spectrometers, Raman spectrometers, and electrochemical workstations to analyze the assay readouts.

The development of a simple, portable, affordable detection system would significantly improve the ease of detection of heavy metal contamination in remote locations or delicate ecosystems. Smartphones are portable, widely available, user-friendly, and therefore well suited to act as an effective platform for on-site detection [27,28]. Many interesting biosensors have been designed based on smartphones utilizing mobile apps and various connected devices, such as high-performance cameras and light sensors [28,29,30,31,32,33,34,35,36]. For example, Wei et al. reported a dual-illumination color and dual-cuvette hand-held detection platform that used a smartphone’s camera and image processing app for monitoring mercury contamination in water [37]. Another group, Ana et al., designed a colorimetric and fluorescence quantitative detection platform based on a smartphone’s cameras and image processing app to detect a prostate-specific antigen (PSA) in a whole blood sample [38]. Wang et al. synthesized nitrogen-doped carbon nanodots and used these nanodots to detect mercury, and the results were produced using a smartphone app [35]. Here, our group developed two detection methods based on a smartphone’s ambient light sensor and light meter app, which can be used for on-site detection of mercury contamination [39,40]. Compared with traditional biosensors, our smartphone-based biosensor is more efficient, simple, and user-friendly, and only a very small volume of sample is used for the assay. This results in a lowered overall cost, making this portable assay easily available for use in on-site tests outside laboratories. We demonstrate that our smartphone-based platform for bioanalysis is widely scalable and has potential additional prospective applications.

To provide an accurate and portable platform to sensitively quantify heavy metal ion in water samples, we report a system here that consists of an Android phone (MI 4) and a lightweight (3.5 g) attachment. The principle is based on using an ambient light sensor of a smartphone to measure mercury-induced subtle transmitted light intensity changes in a gold nanoparticle-based colorimetric assay. The colorimetric assay for Hg^2+^ was first introduced by the Yang group [41]. We demonstrate that our assay is extremely sensitive to mercury in water samples, with a limit of detection (LOD) of ~0.28 ng/mL. This assay can easily detect the maximum contaminant level (MCL) of mercury(II) recommended for drinking water as established by the U.S. Environmental Protection Agency (EPA) and the World Health Organization (WHO) (2 and 1 ng/mL respectively), making it highly useful for determining whether drinking water is safe for consumption [42,43].

## 2. Materials and Methods

### 2.1. Materials and Apparatus

The DNA was synthesized and purified by Shanghai Sangon Biotechnology Co., Ltd. (Shanghai, China), and it had the following sequence: (5’-AAAAAAAAAATTCTTTCTTCCCCTTGTTTGTT-3’). Chloroauric acid (HAuCl_4_) was purchased from Sigma-Aldrich. All aqueous solutions were prepared with double-distilled water (ddH_2_O) (Milli-Q, Millipore, Boston, MA, USA). All other chemicals were of analytical grade and used without any further purification. Light-emitting diodes (LEDs) (510 nm) were purchased from Shenzhen OCtai Co., LTD. (Shenzhen, China). A battery (3 V) was purchased from VSAI. Transparent white plastic pipes with a diameter of 12 mm, and with a resistance of 30,000 Ω, with a switch and wires were collected from a local store. A 20 mM phosphate buffer containing 100 mM KCl (pH 7.0) was used throughout the experiments. UV-vis adsorption spectra were recorded on a synergy H1 hybrid multi-mode microwell reader (Bio-Tek Instruments, Inc., Winooski, VT, USA). ICP-MS (ThermoFisher, Waltham, MA, USA) was also used.

### 2.2. Synthesis of Gold Nanoparticles (AuNPs)

AuNPs (~16 nm diameter) were prepared using a citrate reduction of HAuCl_4_ through an established procedure [44]. Briefly, a HAuCl_4_ solution (2 mL 1% HAuCl_4_) was rapidly added to 100 mL of boiling ddH_2_O with vigorous stirring in a round bottom flask. Subsequently, 4 mL of a 1% sodium citrate solution was rapidly added to the solution. The solution was boiled for 10 min, during which its color changed from pale yellow to wine red, and the reaction mixture was then stored at 4 °C before use. AuNPs were characterized by transmission electron microscopy (TEM) using a PHILIPS TECNAI-10 transmission electron microscope with an acceleration voltage of 120 kV.

### 2.3. Design of the Smartphone-Based Microwell Reader (MR S-Phone) Attachment

The auxiliary device consists of a switch, a 30,000 Ω resistor, three batteries (3 V), and a 520 nm LED. All of these electrical and optical elements were consolidated into a cylindrical plastic housing (diameter = 14 mm; length = 38 mm; weight = 3.5 g). The profile was implemented on a smartphone’s ambient light-sensor (MI-4).

### 2.4. Colorimetric Detection of Hg^2+^

A standard AuNPs-aptamer colorimetric analysis experiment was carried out as follows: 50 μL AuNPs (16 nm in diameter) were mixed with a 3 μM aptamer (which is a single-stranded short oligonucleotide that exhibits high specificity for binding Hg^2+^ in a 20 mM phosphate buffer (pH 7.0) and incubated for 10 min to form the probe solution. Next, 10 μL of water sample solution containing Hg^2+^ (0–128 ng/mL) were added to the probe solution and incubated for 10 min, followed by an addition of 10 μL NaCl (3 M) for colorimetric detection. A photograph was taken, and the TEM images revealed that the morphology of the AuNPs-aptamer was different at different concentrations of Hg^2+^.

### 2.5. Procedure of Hg^2+^ Detection Using the Synergy H1 Hybrid Multi-Mode Microplate Reader and the MR S-Phone System

The typical mercury detection experiment was carried out as follows: First, a 10 μL water sample solution containing Hg^2+^ was added to 50 μL AuNPs-aptamer probe solutions and incubated for 10 min. Next, 10 μL of 3 M NaCl were added to the sample, and the absorption spectrum (400 nm to 760 nm) of the sample, the absorption light intensity (520 nm), and the transmitted light intensity were detected using a synergy H1 hybrid multi-mode microplate reader and the MR S-phone system, respectively.

The procedure for using the MR S-phone system to detect Hg^2+^ is as follows: Following the colorimetric analysis of the water sample, the sample was inserted in a custom-designed attachment (described above). The attachment was then connected to the ambient light sensor on a smartphone. The microwell containing the sample was positioned in the middle of the LED and ambient light sensor. The attachment was activated, and the excitation light from the LED was transmitted through the sample and then measured by the ambient light sensor. Lastly, we used the light meter app to record and display the collected data.

## 3. Results and Discussion

### 3.1. Overview of the Smartphone-Based Microwell Reader (MR S-Phone) Attachment

**Figure 1:**
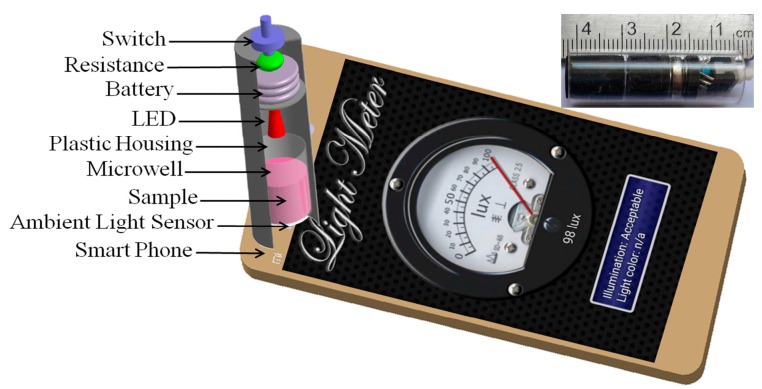
Schematic illustration of the microwell reader installed on an Android-based smartphone and image of the actual optical microwell reader.

We developed a battery-powered attachment that can be connected to a smartphone’s ambient light sensor to quantify the mercury concentration in aqueous samples using a colorimetric aptamer nanosensor. This device is comprised of a switch, which was used to control the instruments; a 10,000 Ω resistance, which was used to adjust light intensities; three button batteries (3 V), which were used to power the LED; and the LED (520 nm), which was used to provide a stable light source. In this colorimetric aptamer nanosensor for Hg^2+^, the maximum absorption wavelength of the samples was determined to be 520 nm. Therefore, we selected an LED with an emission wavelength of 520 nm. These components were assembled in a cylindrical plastic housing (diameter = 12 mm; height = 42 mm) with a total weight of 3.5 g. The water samples were contained in a microwell with black walls and a transparent bottom. This microwell was selected because it can eliminate the ambient light interference, ensuring the ambient light sensor of the smartphone only receives light from the LED in the attached device (Figure 1).

### 3.2. The Principle of the MR S-Phone-Based Hg^2+^ Colorimetric Detection System

**Figure 2:**
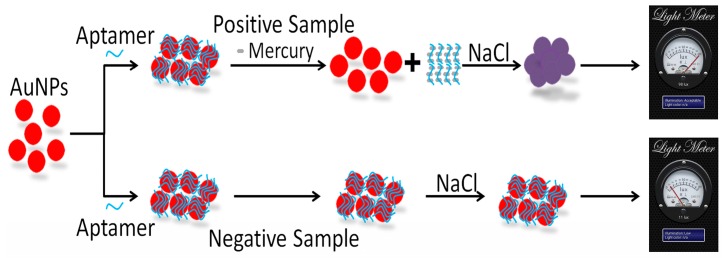
Schematic illustration of the workflow for the smartphone-based microwell reader (MR S-phone)-based Hg^2+^ colorimetric detection system. In the absence of heavy metal contaminants (lower), the aptamer adsorbs to AuNPs, which protects the AuNPs from aggregation induced by the addition of high-concentration salt. In Hg^2+^-positive samples, the aptamers are competed away from AuNPs by Hg^2+^. Therefore, when the solution is treated with NaCl, the AuNPs aggregate, and the solution changes to blue. The light meter app is then used in combination with the phone attachment to detect the transmitted light intensity of each sample. When Hg^2+^ is present, the MR S-phone system detects an increase in transmitted light intensity. When samples lack Hg^2+^, the AuNPs are protected by the aptamers in high-concentration salt; therefore, the solution remains red, and no change in transmitted light intensity is detected.

A diagram of the MR S-phone-based Hg^2+^ colorimetric detection system is displayed in Figure 2. First, the synthesized AuNPs in aqueous solution are stable: AuNPs aggregate in a high salt solution due to a net change in surface charge, and the solution color will change from red to purple or blue [45,46]. The aptamer, which is a single-stranded DNA oligonucleotide, adsorbs to the surface of the AuNPs, protecting them from aggregating in a high salt solution, and the solution retains its characteristic red color. When Hg^2+^ is present in the sample, the aptamer will preferentially interact with Hg^2+^. This results in aptamer desorption from the AuNPs, further resulting in AuNP aggregation and sample color changing from red to purple or blue. After the colorimetric assay was completed, the microwell containing the sample was put into the MR S-phone attachment, and the assay liquid was then put into the MR S-phone attachment. Then, the MR S-phone attachment was connected to the ambient light sensor of the smartphone to produce a readout of the results. For a positive sample control, NaCl (3 M) was added to the AuNPs without any aptamer present, the reaction solution changed to blue, and the transmitted light intensity increased as determined using the ambient light sensor. For a negative control sample, AuNPs were not treated with NaCl, the color of the reaction solution was red, and the transmitted light intensity of the sample was unchanged. Subsequently, a series of mercury concentrations were tested, and the results indicated that the transmitted light intensities of the samples scaled linearly with the concentration of Hg^2+^.

### 3.3. AuNP-Based Aptamer-Assay for Detection of Hg^2+^

**Figure 3:**
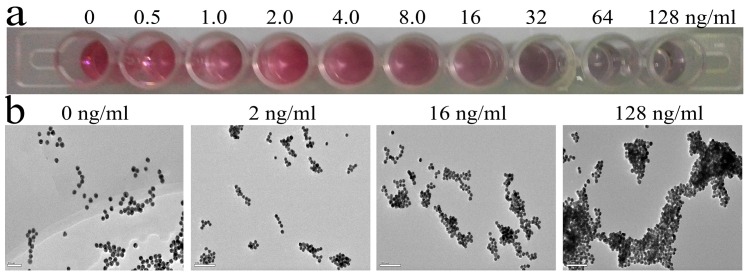
The characterization of AuNP-based aptamer-assay for detection of Hg^2+^. (**a**) Image of the assay colorimetric change with different concentrations of Hg^2+^; (**b**) TEM images of AuNPs mixed with a 3 μM aptamer and 3 M NaCl, in the presence of various concentrations of Hg^2+^.

The colorimetric assay is based on the principle that the characteristic color of the AuNPs-aptamer solution will change from red to purple when NaCl is added to a sample containing Hg^2+^. As shown in Figure 3a, a clearly distinguishable color change from brilliant red to light purple can be observed with the naked eye. When the Hg^2+^ concentration increased from 0 to 128 ng/mL, the reaction solutions gradually turned purple. The TEM images revealed that the morphology of the AuNPs (~16 nm) gradually changed from mono-dispersion to aggregation with increasing concentrations of Hg^2+^ (0 ng/mL, 2 ng/mL, 16 ng/mL, and 128 ng/mL) and 3 M NaCl (Figure 3b). As mentioned above, the degree of aggregation depends on the Hg^2+^ concentration.

### 3.4. The Performance of the MR S-Phone System for Hg^2+^ Detection

The Hg^2+^-specific aptamer can be adsorbed on AuNPs, which protects AuNPs from aggregation induced by the addition of high-concentration NaCl. Different concentrations of Hg^2+^ from 0–128 ng/mL were added to the prepared AuNPs-aptamer, and the absorption spectrometry and transmitted light intensity were measured using the synergy H1 hybrid multi-mode microwell reader and the MR S-phone system, respectively.

**Figure 4:**
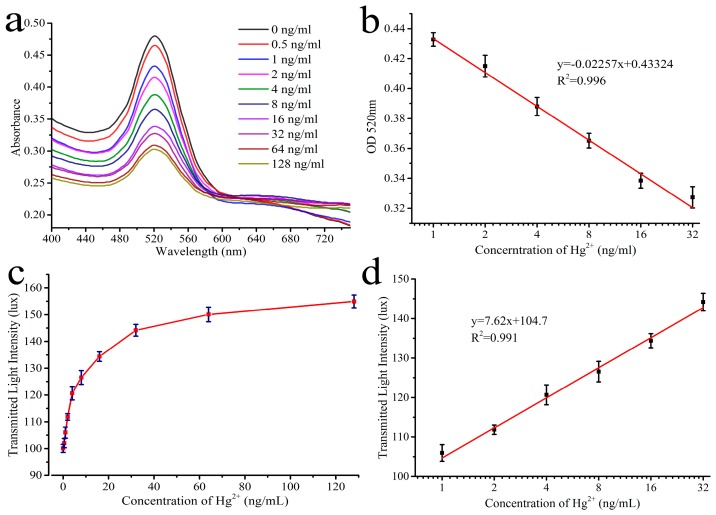
The performance of the MR S-phone system for detection of Hg^2+^ in water. (**a**) UV-vis spectroscopy based on the colorimetric detection of different concentrations of Hg^2+^; (**b**) Calibration curve of colorimetric assay for detecting Hg^2+^ using the intensity of the 520 nm peak; (**c**) Transmitted light intensity of the MR S-phone system with different concentrations of Hg^2+^; (**d**) The calibration curve of the MR S-phone system for detection of Hg^2+^. Each value represents the mean of three independent experiments (*n* = 3).

The UV-vis spectroscopy (Figure 4a) was used to explore the change in AuNP aggregation induced by Hg^2+^ in this system. The dispersed AuNPs displayed a surface plasmon peak at 520 nm. Upon aggregation, the intensity of the 520 nm peak decreased. Next, a standard Hg^2+^ concentration curve was used to determine the linear range of the spectroscopic assay (Figure 4b). The linear range of the assay was determined to be 1–64 ng/mL with a correlation of 0.996, exhibiting an excellent sensitivity.

To evaluate the MR S-phone system, Hg^2+^ samples at various concentrations were tested, and the results were analyzed using the light meter app. As shown in Figure 4c, the transmitted light intensity increased with increasing Hg^2+^ concentration. Using a standard curve of Hg^2+^ with the transmitted light intensity assay, the LOD for the Hg^2+^ colorimetric assay was determined to be 0.28 ng/mL, and the linear detection range was from 1 ng/mL–32 ng/mL with a correlation of 0.991 (Figure 4d).

### 3.5. The Selectivity of the MR S-Phone System

**Figure 5:**
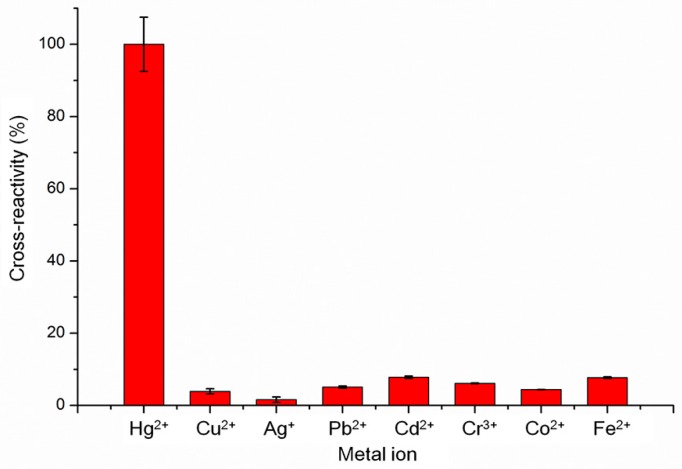
The selectivity of the MR S-phone detection method. The MR S-phone system was used to detect the presence of other metal ions (Cu^2+^, Ag^+^, Pb^2+^, Cd^2+^, Cr^3+^, Co^2+^, and Fe^2+^). Each value represents the mean of three independent experiments (*n* = 3).

In order to evaluate the specificity of the sensor in detection of Hg^2+^, the cross-reactivity was also explored using other metal ions, including Cu^2+^, Ag^+^, Pb^2+^, Cd^2+^, Cr^3+^, Co^2+^, and Fe^2+^, and the cross-reactivity rate was normalized to Hg^2+^ reactivity. As shown in Figure 5, the cross-reactivity rates of Cu ^2+^, Ag^+^, Pb^2+^, Cd^2+^, Cr^3+^, Co^2+^, and Fe^2+^ were less than 10% using the AuNPs-aptamer smartphone mercury assay, suggesting that this system has a high specificity for detecting Hg^2+^. However, most real-world systems consist of mixtures of heavy metal ions; therefore, we also detected the mixtures samples (Figure S1). The result demonstrates high selectivity using the MR S-phone system.

### 3.6. Analysis of Samples

The feasibility of the MR S-phone system for the analysis of spiked Hg^2+^ tap water samples and Pearl River samples (Guangzhou, China) was validated. The analytical results are shown in Table 1. The recoveries for all these cases were 93%–113%, suggesting that the MR S-phone system has a relatively high recovery.

## 4. Conclusions

We developed a novel aptamer colorimetric assay for the detection of mercury (Hg) contamination in aqueous samples, which employs a novel detector device based on a smartphone. The linear detection range of this method for Hg^2+^ was 1 to 32 ng/mL, and the LOD was 0.28 ng/mL. The strategy presented here has several significant advantages: first, the method is simple, cheap, portable, and easy to operate; second, the method has high sensitivity and selectivity for the detection of mercury. Our newly established method can significantly improve and simplify tests for on-site assessment of mercury contamination in ground water and drinking water. This strategy could also be potentially adapted for the detection of other heavy metal contaminants in the environment.

## Figures and Tables

**Table 1:** Detection of Hg^2+^ in tap water samples and Pearl River samples (Guangzhou, China) using the MR S-phone system.

Sample	Spiked Concentrations (ng/mL)	Result of ICP-MS (ng/mL)	Recovery of ICP-MS (%)	Result of the MR S-Phone (ng/mL)	Recovery of the MR S-Phone (%)	Coefficient of Variation (%)
Tap Water	1	0.98	98	0.97 ± 0.01	97	1.0
	10	10.1	101	11.4 ± 0.06	113	0.5
	20	21	105	18.6 ± 0.17	93	0.9
	30	29.7	99	30.8 ± 0.15	102	0.5
Pearl River	2	1.9	95	2.2 ± 0.1	110	4.5
	13	12.4	95.4	13.8 ± 0.15	105	1.1
	22	22.3	101	22.3 ± 0.2	101	0.9
	31	30.8	99.4	31.1 ± 0.25	103	0.8

## Supplementary Materials

The following are available online at http://www.mdpi.com/1424-8220/16/11/1871/s1. Figure S1: The selectivity of the MR S-phone detection method.
