# Supplementary material for: A Portable Smart-Phone Readout Device for the Detection of Mercury Contamination Based on an Aptamer-Assay Nanosensor
Source: Sensors (Basel). 2016 Nov 8;16(11):1871. doi: 10.3390/s16111871 (PMC5134530; doi:10.3390/s16111871)
Supplement: Supplementary file 1 [file sensors-16-01871-s001.pdf]

Supplementary Materials: A Portable Smart-Phone Readout Device for the Detection of Mercury Contamination Based on an Aptamer-Assays Nanosensor

Wei Xiao, Meng Xiao, Qiangqiang Fu, Shiting Yu, Haicong Shen, Hongfen Bian and Yong Tang

Most real-world systems consist of mixtures of heavy metal ions; therefore, we performed an experiment to detect the mixtures samples of Hg^{2+} and other ions. Interference effects of 50 ng/mL levels of various metal ions (Fe^{2+}, Ag^{+} and Pb^{2+}) were added to 10 ng/mL Hg^{2+} solutions. The result demonstrates high selectivity using the smartphone-based microwell reader (MR S-phone) system (Figure S1).

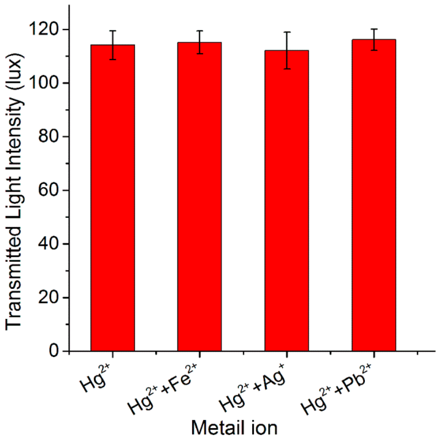

Figure S1. The selectivity of the smartphone-based microwell reader (MR S-phone) detection method.
